# Multivalent COBRA Hemagglutinin and Neuraminidase Influenza Vaccines Adjuvanted with TLR9 Agonist CpG 1018

**DOI:** 10.3390/vaccines13070662

**Published:** 2025-06-20

**Authors:** Pedro L. Sanchez, Amanda Lynch, Ted M. Ross

**Affiliations:** 1 Center for Vaccines and Immunology, University of Georgia, Athens, GA 30602, USA; sanchep3@ccf.org; 2 Florida Research and Innovation Center, Cleveland Clinic, Port Saint Lucie, FL 34987, USA; lyncha9@ccf.org; 3 Department of Infection Biology, Lerner Research Institute, Cleveland Clinic, Cleveland, OH 44195, USA; 4 Department of Infectious Diseases, College of Veterinary Medicine, University of Georgia, Athens, GA 30602, USA

**Keywords:** influenza, hemagglutinin, neuraminidase, TLR9, COBRA

## Abstract

Background/Objectives: There is a need for effective seasonal influenza virus vaccines that provide broad and long-lasting protection against influenza virus infections. Methods: In this study, next-generation influenza hemagglutinin (HA) and neuraminidase (NA) vaccine candidates designed using the computationally optimized broadly reactive antigen (COBRA) methodology were formulated with the TLR9 agonist, CpG 1018. These adjuvanted COBRA HA/NA vaccines were administered intramuscularly or intranasally to mice with pre-existing anti-influenza immunity or immunologically naïve mice. Results: Mice with pre-existing immune responses to historical influenza virus strains vaccinated intranasal (IN) with COBRA HA/NA vaccines adjuvanted with CpG 1018 had enhanced IgG titers in their bronchoalveolar lavages (BALF) compared to unadjuvanted vaccines. These mice also had increased serum IgG titers that were like antibody titers observed in mice that were vaccinated intramuscularly. Mice that were vaccinated intranasally with this adjuvanted vaccine also had antibodies with significantly higher hemagglutination inhibition activity against a broad range of H1N1 and H3N2 influenza viruses and more HA and NA specific antibody-secreting cells compared to unadjuvanted vaccine. Following the H1N1 influenza virus challenge, pre-immune mice that were vaccinated with the COBRA HA/NA vaccine with CpG 1018 were protected from morbidity and mortality and had no detectable viral lung titers. Conclusions: Overall, CpG 1018 adjuvanted COBRA HA/NA elicited enhanced protective antibodies compared to the unadjuvanted vaccine against several drifted H1N1 and H3N2 influenza viruses in pre-immune mice that were either intramuscularly or intranasally vaccinated with a balanced Th1/Th2 immune response.

## 1. Introduction

Influenza A viruses belong to the family *Orthomyxoviridae* and cause respiratory infections. In the United States, it is estimated that 5–20% of individuals developed seasonal influenza infections that caused mild to severe symptoms, including death, and resulted in ~290,000 to 650,000 annual mortalities worldwide [1,2]. Influenza viruses have several mechanisms to evade the host immune response, including antigenic drift and shift, where the virus particles undergo changes of their surface glycoproteins hemagglutinin (HA) and neuraminidase (NA), as these two proteins are the primary targets of antibodies elicited by influenza viruses. Since influenza viruses are always evolving to escape immune responses elicited by natural infection or vaccination, vaccines are updated annually for each hemisphere. However, yearly vaccines are not always well-matched to the circulating virus strains during a given season, leading to decreased vaccine efficacy [3]. Current commercial vaccines are formulated as either split-inactivated viruses, purified subunit proteins, or live-attenuated virus vaccines [1,4]. To enhance the effectiveness of licensed vaccines, adjuvants, such as the oil-in-water emulsion MF59, are formulated with influenza virus vaccines to induce protective immune responses in high-risk populations, including the elderly [5,6].

In an attempt to develop an improved next-generation influenza vaccine, our group has designed HA and NA molecules using a computationally optimized broadly reactive antigen (COBRA) methodology [7,8]. These HA and NA antigens elicit antibodies with hemagglutination inhibition (HAI) and neuraminidase inhibition (NAI) activity against historical and contemporary influenza A and B viruses in several animal models [7,9,10,11]. In this study, CpG 1018 was used as an adjuvant and formulated with these next-generation COBRA hemagglutinin and neuraminidase recombinant proteins. Mice with pre-existing immunity to influenza or immunologically naïve mice were vaccinated intramuscularly or intranasally with these adjuvanted vaccine formulations.

Toll-like-receptor (TLR9) agonists, such as CpG oligodeoxynucleotides, enhance immune responses generated by influenza virus vaccines [12]. CpG 1018 is a short 22-mer oligonucleotide sequence containing CpG motifs found in rodents, non-human primates, and humans and has been licensed for use in a hepatitis B virus vaccine, HEPLISAV-B^®^ [13]. TLR9 agonists, such as microbial DNA or synthetic CpG oligonucleotides, act via the activation of TLR9 pattern recognition receptors (PRRs) that are located intracellularly within endosomes inside of immune cells, such as dendritic cells, B cells, and macrophages [14,15]. In turn, the activation of TLR9 by CpG 1018 induces the production of pro-inflammatory responses, such as IFN-γ. However, an intranasal route of vaccination may induce mucosal and systemic immune responses that enhance protective immunity in individuals with weak immune systems [16]. Overall, COBRA HA and NA proteins, mixed with CpG 1018 and administered intranasally to mice, enhanced broadly reactive serum and mucosal antibodies that protected mice against lethal influenza virus infection compared to mice vaccinated with unadjuvanted COBRA-based vaccines.

## 2. Materials and Methods

### 2.1. Antigen Construction and Synthesis

The Y2 (H1) and NG2 (H3) COBRA hemagglutinin (HA) and N1-I and N2-B COBRA neuraminidase (NA) proteins were designed using a next-generation COBRA methodology as previously described [7,9,10,11]. The recombinant COBRA HA and NA proteins were purified from HEK 293T cells transfected with pcDNA3.1 plasmids expressing a truncated HA with T4 foldon domain for trimerization as well as the addition of an Avitag and 6x HIS tag as previously described [17]. The concentrations of the purified rHA proteins were determined using BCA (Thermo Fisher, Waltham, MA, USA).

### 2.2. Vaccination and Infection

**Figure 1:**
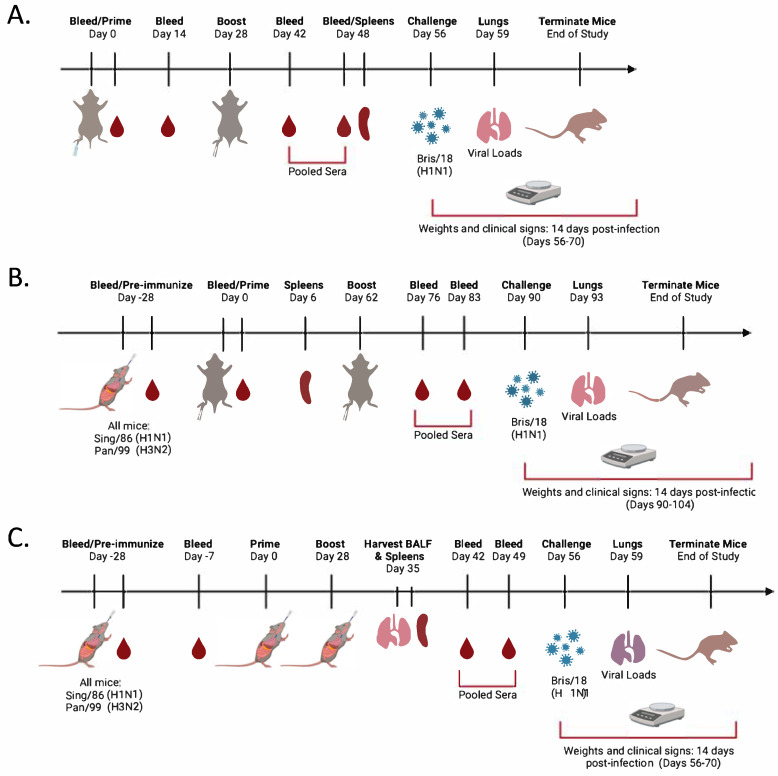
Schematic of the study timelines. DBA/2J (6–8 weeks old) female mice were randomly divided into 21 groups. (**A**) 9 naïve groups with n = 18 mice were vaccinated intramuscularly (IM) with 12 mg of each COBRA HA (Y2, NG2) and COBRA NA (N1-I, and N2-B) proteins plus the addition of 10 mg of CpG or no adjuvant. All naïve mice were bled and vaccinated on day 0, which was followed by blood collection on day 14. Mice were vaccinated again on day 28. On days 42 and 48, mice were bled, and spleens were harvested from 4 mice from each group on day 48. On day 56, all mice were intranasally challenged with the H1N1 influenza virus BR/18. Three days post-infection, lungs were harvested from 4 mice from each group, and the remainder of the mice were monitored for 14 days post-infection. (**B**) Mice were infected intranasally with the historical H1N1 Sing/86 and H3N2 Pan/99 influenza viruses at 2.5^105 PFU/25 μL of each virus for a total 50 μL dose per mouse. All these mice were vaccinated IM (**B**) or IN (**C**) with 12 mg of COBRA HA and NA proteins (Y2, NG2, N1-I, and N2-B) plus the addition of 10 mg of CpG alone or no adjuvant. As controls, some of the mice were vaccinated with COBRA HA/NA mixture (12 mg) or mock vaccinated. All pre-immune mice were bled at day −28, at day −7 (IN groups), or at day 0 (IM groups) and vaccinated on day 0, followed by a second vaccination on day 62 (IM groups) or day 28 (IN groups). BALFs and spleens (IN groups) were harvested from two mice from each group at 7 days post-boost (day 35), and four spleens were harvested from four mice (IM groups) at day 6 following the first vaccination. On days 76 and 83 (IM groups) or 42 and 49 (IN groups), all mice were bled and then intranasally infected (Day 90 or 56) with Bris/18. Three days post-infection, lungs were harvested from three mice from each group, and the remaining mice were monitored for 14 days post-infection. Created with BioRender.com.

Six-to-eight-week-old DBA/2J female mice (n = 128); Jackson Laboratory (Bar Harbor, ME, USA) were cared for and approved by the Institutional Animal Care and Use Committee (IACUC) (no. A2020 03-007-Y1-A0 and LRI2935 approved on 24 August 2022). Mice were randomly divided into subsets according to routes of administration, intramuscular (IM) (Figure 1A,B) or intranasal (IN) (Figure 1C). They were divided into three vaccine groups (IM naïve) with an n = 18 per group, three groups (IM pre-immune) with an n = 18 per group, or two groups (IN pre-immune) with an n = 10 per group. Prior to vaccination (day 0), all of the mice were bled via the submandibular vein to confirm the absence of antibodies specific to seasonal H1N1 influenza viruses, including: A/Singapore/06/1986 (Sing/86), A/Brisbane/59/2007 (Bris/07), A/California/07/2009(Cal/09), A/Brisbane/02/2018 (Bris/18), A/Guangdong/SWL1536/2019 (GD/19) and H3N2 viruses: A/Panama/2007/1999 (Pan/99), A/Switzerland/9715293/2013 (Swit/13), A/HongKong/4801/2014 (HK/14), A/Singapore/IFNIMH/2016 (Sing/16), A/Kansas/14/2017(KS/17), A/HongKong/2671/2019(HK/19), and A/South Australia/34/2019 (SA/19). On day −28, mice predestined for pre-immunity (Figure 1B,C) were anesthetized with 2–3% isoflurane and received IN distillation with 50 μL of 2.5 × 10^5^ PFU of Sing/86 H1N1 and 2.5 × 10^5^ PFU of Pan/99 H3N2 influenza A viruses (IAVs). Upon recovery, the mice were returned to their cages and monitored daily for up to 14 days post-infection for weight loss, clinical signs, and survival. On day −7, some of the mice were bled, and the rest were allowed to rest for the remainder of the 28 days (Figure 1). After 28 days (Day 0; IM and IN pre-immune mice) or at day 0 (IM naïve mice), all of the mice were vaccinated either IM via the hindleg or IN via the nares (Figure 1) with 50 μL of vaccine formulations containing 3 μg of each of the COBRA rHA and rNA proteins, Y2, NG2, N1, and N2 (COBRA HA/NA), in cold tris buffer solution and adjuvanted with 10 μg of CpG 1018. As controls, some mice were vaccinated with 50 μL of COBRA HA/NA vaccines without adjuvant (tris buffer only), mock vaccinated with 50 μL of tris buffer only (no vaccine or adjuvant; pre-immunity only), or vaccinated with CpG 1018 alone (tris buffer plus CpG 1018; naïve mice only). Following vaccination, all mice were placed back into their cages and monitored. On day 14 post-vaccination, all naïve mice were bled (Figure 1A), and sera were separated from blood cells via centrifugation and then stored at −20 °C ± 5 °C. On day 28 (IM naïve and IN pre-immune) (Figure 1A,C) or day 62 (IM pre-immune) (Figure 1A), all mice were boosted as before, following the same vaccination regimens and routes of administration. Six days (IM pre-immune mice) or 20 days (IM naïve mice) post boost (Figure 1A,B), some of the mice from each group were euthanized, and spleens were harvested, homogenized, and stored in 90% fetal bovine serum (FBS) plus 10% dimethyl sulfoxide (DMSO) solution at −80 °C for 24 h and then moved to liquid nitrogen (LN). Concurrently, at 7 days (IN pre-immune mice) post boost (Figure 1C), some of the mice were euthanized and spleens harvested and processed as before, as well as harvest of lung bronchoalveolar lavage fluids (BALFs). Briefly, the lungs were flushed via the trachea with 450 μL of cold 1× PBS followed by centrifugation at 3000× *g* for 5 min, and the samples were stored at −20 °C ± 5 °C. Following the boost vaccinations (after 14 and 21 days), all of the DBA/2J mice were bled on days 42 and 48 (IM naïve mice), 42 and 49 (IN pre-immune mice), or 76 and 83 (IM pre-immune mice), as before (Figure 1A,B), and the collected sera was separated at 10,000 rpm for 10 min and both corresponding serum timepoints pooled together equally and then stored at −20 °C ± 5 °C. At day 56 (IM naïve and IN pre-immune) or day 90 (IM pre-immune), all mice were anesthetized with 2–3% isoflurane and received IN distillation with 50 μL of 8 × 10^6^ PFU of Bris/18 (H1N1) influenza A virus (Figure 1) and monitored daily for 14 days for morbidity and mortality. At day 59 (IM naïve and IN pre-immune) or day 93 (IM pre-immune), lungs were harvested and stored at −80 °C (Figure 1).

### 2.3. Enzyme-Linked Immunosorbant Assay (ELISA)

Total IgG antibody reactivity in serum or BALFs against Bris/18 (rHA), Bris/18 (rNA), Sing/16 (rHA), or Swit/13 (rNA). The optical density (O.D.) of the samples was immediately read at 414 nm in a spectrophotometer (PowerWave XS, BioTek, Santa Clara, CA, USA) using the Gene05 software (version 3.14, https://www.agilent.com/en/support/biotek-software-releases (accessed on 4 April 2024)) to measure the antibody endpoint titers and compared to positive and negative controls. In addition, IgA, IgG1, IgG2a, and IgG2b isotype binding to HA and NA proteins were also assessed. Lung lavages were prepared at an initial 1:10 and serially diluted (1:2) for detection of IgA, IgG1, IgG2a, and IgG2b.

### 2.4. Hemagglutination Inhibition Assay (HAI)

The HAI assay was performed for the detection of sera antibodies that inhibit influenza viruses from agglutinating red blood cells (RBCs) by preventing the binding of viral surface HA to sialic acid residues on RBCs. The HAI assays were performed against a panel of H1N1 viruses, including: Sing/86, Bris/07, Cal/09, Bris/18, GD/19, and H3N2 viruses: Pan/99, Swit/13, HK/14, Sing/16, KS/17, HK/19, and SA/19 as previously described [18]. Sera from each mouse was initially treated with receptor destroying enzyme (RDE) (Denka Seiken, Co., Tokyo, Japan) to eliminate non-specific inhibitors and heat-inactivated in a water bath at 56 °C for 45 min. The RDE-treated sera was serially diluted, and each virus was prepared at 8 HA units and incubated at RT for 20 min for H1N1 influenza viruses or 30 min for H3N2 influenza viruses. Following incubation, 0.8% turkey red blood cells (TRBCs) for H1N1 viruses or guinea pig red blood cells (GPRBCs) for H3N2 influenza viruses were added and then incubated at RT for 30 min (H1N1) or 1 h (H3N2). After incubation, the titer of each serum sample was reported as the reciprocal dilution of the last well without agglutination.

### 2.5. Enzyme-Linked Lectin Assay (ELLA)

To assess the ability of vaccinated mice serum to inhibit influenza-specific NA enzymatic activity as previously described [16]. Briefly, 100 μL of a 25 μg/mL fetuin (Sigma-Aldrich, St. Louis, MO, USA) in KPL coating buffer (Seracare Life Sciences Inc, Milford, MA, USA) was incubated overnight and stored at 4 °C until ready for use. Mouse sera samples were serially diluted 2-fold from an initial 1:10 dilution in sample diluent. Fifty microliters of 2-fold serial dilutions of rNA proteins and 50 μL of the serially diluted serum samples were added into the fetuin-coated plate containing 50 μL of sample diluent in duplicate. Plates were incubated for 18 h at 37 °C, and then 100 μL of peanut agglutinin-HRPO (Sigma-Aldrich, St. Louis, MO, USA) diluted 1000-fold in conjugate diluent (DPBS, 1% BSA) was added to each well, followed by 2 h incubation at RT. After the incubation, o-phenylenediamine dihydrochloride (OPD) (500 μg/mL) (Sigma-Aldrich, St. Louis, MO, USA) in 0.05 M phosphate-citrate buffer with 0.03% sodium perborate pH 5.0 (Sigma-Aldrich, St. Louis, MO, USA) solution was added and incubated in the dark for 10 min at RT. The colorimetric reaction was stopped by adding 100 μL of 1 N sulfuric acid per well. The absorbance was read at 490 nm using a spectrophotometer (PowerWave XS, BioTek, Santa Clara, CA, USA) utilizing the Gen05 software (version 3.14, https://www.agilent.com/en/support/biotek-software-releases (accessed on 4 April 2024)). The NA activity was determined after subtracting the mean background absorbance of the negative-control wells and dividing by the mean of the positive-control wells. Linear regression analysis was used to determine the dilutions of the Swit/13 and Bris/18 rNAs used in the assay necessary to achieve 90 to 95% NA activity and was used for determining the Log_2_ 50% titer of the NA inhibition enzyme-linked lectin assays (ELLAs).

### 2.6. Lung Viral Titers

Viral titers from collected lungs post-infection were assessed by plaque assay as previously described [11]. MDCK cells (1 × 10^6^ cells per 10 cm^2^) were incubated for 24 h and grown to ~95% confluency. Day 59 (IM naïve), day 93 (IM pre-immune), or day 56 (IN pre-immune) lungs from each mouse were weighed and homogenized, and the diluted samples were then added to the MDCK monolayers. After one hour of incubation, samples were exposed to 15 min shaking intervals at RT. Following 1 h, the media was removed, and the cells were exposed to a 1:1 solution of 1.6% agarose in 2× cMEM media containing TPCK-Trypsin at 1 μg/mL and incubated for 2–5 days. Once cytopathic effects were confirmed, the agarose layers were removed from each well, and the cells were fixed with 10% formalin solution for 10 min at RT. Following formalin removal, the cells were stained with 1% crystal violet (Fisher Science Education, Waltham, MA, USA), the plates were allowed to dry, and the plaque-forming units (PFUs) were counted, followed by calculation of the lung viral titers as PFU/g of tissue.

### 2.7. FluoroSpot Assay

At day 48 (IM naïve), day 6 (IM pre-immune), or day 35 (IN pre-immune), spleens were collected (n = 3 for IM naïve and IM pre-immune, or n = 2 for IN pre-immune) per study arm. Splenocytes were assessed for antigen-specific antibody-secreting cells (ASCs) using the two-color Immunospot^®^ kit (CTL, Shaker Heights, OH, USA). As indicated in the manufacturer’s instructions, day 48 (IM naïve) resting splenocytes were washed with media and filtered through 70 μm MACS^®^ SmartStrainers to remove debris and resuspended in media containing Poly-S for 5-day stimulation. Similar processing was performed for pre-stimulated splenocytes (day 6 and day 35 spleens). In v-bottom, 96-well plates, splenocytes were serially diluted 3-fold, in duplicates, starting at 1 × 10^5^ or 3 × 10^5^ live cells per well and transferred to pre-treated (with 70% ethanol) PVDF plates, coated with anti-Igκ/λ capture antibody or Bris/18 HA at 25 μg/mL. Splenocytes were incubated in a humidified chamber with 5% CO_2_ for 16–18 h at 37 °C. Following incubation, the ASCs were removed from the plates, and the plates were washed twice with 1× PBS. An anti-mouse detection solution containing IgG/IgA was prepared and added to each well of splenocytes and allowed to incubate in the dark for 3 h at RT. After incubation, the plates were washed and dried overnight in the dark. FluoroSpot were detected on the ImmunoSpot^®^ S6 Ultimate Analyzer. Spot-forming units (SFUs) were enumerated using the Basic Count mode of the CTL ImmunoSpot SC Studio (Version 1.6.2, Shaker Heights, OH, USA).

#### Statistical Analysis

All data are presented as mean ± standard error of the mean (SEM). One-way ANOVA was used to analyze HAI, ELISPOT, and lung virus titers between vaccine groups. Two-way ANOVA was used to analyze the total IgG antibody titers for each vaccine group. Weight loss, survival, and clinical scores following viral challenge were also analyzed using two-way ANOVA. Statistical analysis was performed using GraphPad Prism 9 software (GraphPad, San Diego, CA, USA). All *p* values are listed in the figure legend of each figure.

## 3. Results

### 3.1. CpG 1018 Adjuvant Enhances Serum and BALF Anti-HA/NA Antibodies

**Figure 2:**
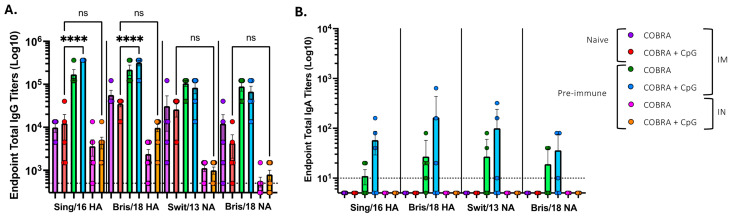
Anti-HA IgG in intramuscularly vaccinated naïve and pre-immune mice. Sera was collected following the second vaccination at days 42 and 48 from each mouse and then pooled. (**A**) Serum samples from naïve mice vaccinated intramuscularly (IM) or intranasally (IN) with or without CpG adjuvant were assessed for total anti-HA IgG binding against Sing/16 (H3) or Bris/18 (H1) rHA, or Swit/13 (H3) or Bris/18 (H1) rNA. (**B**) Serum samples from pre-immune mice vaccinated intramuscularly (IM) or intranasally (IN) with or without CpG adjuvant were assessed for total anti-HA IgA binding against Sing/16 (H3) or Bris/18 (H1) rHA, or Swit/13 (H3) or Bris/18 (H1) rNA. Pre-immune serum anti-influenza total IgA against Sing/16 and Bris/18 rHA, or Swit/13 and Bris/18 rNA. Dotted line represents the level of detection. Each bar represents the average +/− standard error of the mean (SEM). *p* < 0.0001 ****.

Serum and BALF were collected after the vaccine boost and assessed for anti-HA and anti-NA antibodies (Figure 2). Naïve mice vaccinated intramuscularly with COBRA HA/NA vaccine with or without adjuvant had an average ~1:1 × 10^4^ serum anti-HA IgG titer against the Sing/16 H3 HA (Figure 2A) and an average anti-HA IgG titer of ~1:4 × 10^4^ against the Bris/18 H1 HA proteins. In naïve mice vaccinated with COBRA HA/NA proteins mixed with CpG 1018, there was no statistical rise in IgG titers compared to mice vaccinated with only the COBRA HA/NA proteins without adjuvant. Similar results were observed against the N1 and N2 IgG binding antibodies. In contrast to naïve mice, mice with pre-existing anti-influenza immunity to H1N1 and H3N2 influenza viruses had 1–1.5 logs higher anti-HA antibody binding following vaccination. Pre-immune mice that were vaccinated with the same vaccines intranasally had low anti-HA and anti-NA IgG binding titers, regardless of whether the vaccines were formulated with CpG 1018. Only 2–3 pre-immune mice that were vaccinated intramuscularly had IgA-binding antibodies against the HA and NA proteins (Figure 2B).

**Figure 3:**
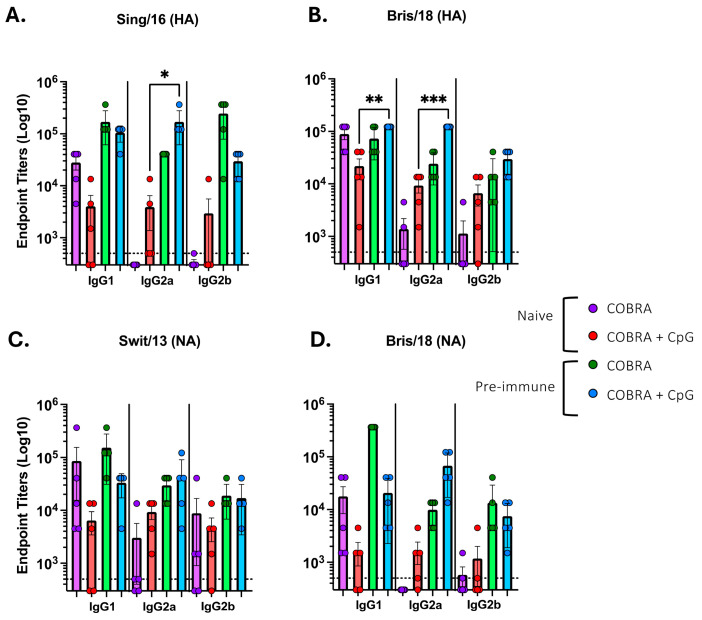
Serum IgG isotypes from naïve mice or pre-immune mice vaccinated intramuscularly. Mice were vaccinated with COBRA HA and NA vaccines (Y2, NG2, N1-I, and N2-B) with CpG. Sera was collected following the second vaccination at days 42 and 48 from each mouse and then pooled. (**A**) Serum samples naïve mice vaccinated intramuscularly (IM) with or without CpG adjuvant and were assessed for IgG1, IgG2a, or IgG2b binding against (**A**) Sing/16 (H3) or (**B**) Bris/18 (H1) rHA, or (**C**) Swit/13 (H3) or (**D**) Bris/18 (H1) rNA. Dotted line represents the level of detection. Each bar represents the average +/− standard error of the mean (SEM). *p* < 0.1 *; *p* < 0.01 **; *p* < 0.001 ***.

Pre-immune mice that were vaccinated intranasally with COBRA HA/NA vaccines mixed with CpG 1018 showed a trend to higher IgG titers in the BALF against both HA and NA proteins compared to mice vaccinated with COBRA vaccines without adjuvant. Naïve mice vaccinated with the COBRA HA/NA recombinant proteins with no adjuvant had a predominately IgG1 isotype profile with little or no detectable IgG2a or IgG2b (Figure 3A,D).

**Figure 4:**
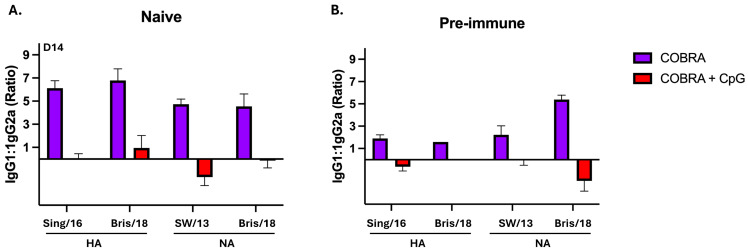
Serum IgG1:IgG2a ratios in naïve and pre-immune mice following intramuscular vaccination. Mice were vaccinated with COBRA HA and NA vaccines with CpG1018 or no adjuvant. Sera were collected following the second vaccination at days 42 and 48 from naïve mice and days 76 or 83 from pre-immune mice. The proportion of serum IgG1 and IgG2a against Sing/16 rHA, Bris/18 rHA, Swit/13 rNA, or Bris/18 rNA is depicted from vaccinated (**A**) naïve mice or (**B**) pre-immune mice. The y-axis indicates the isotype ratios on a Log2 scale, with 0 representing equal IgG1 to IgG2a antibody titers. Each bar represents the average of five individual mice +/− standard error of the mean (SEM).

However, mice vaccinated with the same vaccines plus CpG 1018 had a more mixed IgG isotype response, with all three isotypes detected in almost all mice. Pre-immune mice that were vaccinated with these vaccines with or without CpG 1018 adjuvant had similar mixed isotype profiles. The IgG1:IgG2a ratios were highly biased towards IgG1 in all naïve mice vaccinated with no adjuvant, but all mice vaccinated with COBRA HA/NA vaccines plus CpG had a ratio close to 0, which supports the idea of a more balanced response (Figure 4A,B).

**Figure 5:**
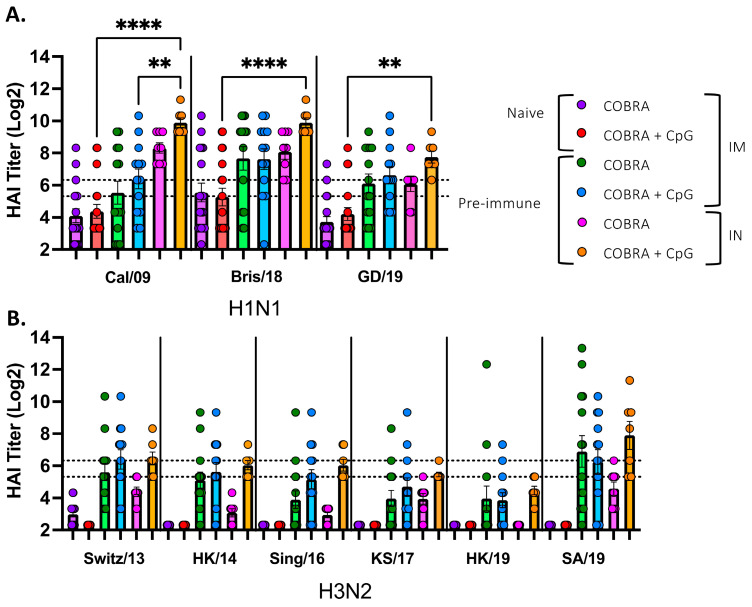
Serum hemagglutination inhibition activity in naïve and pre-immune vaccinated mice. Mice were vaccinated with COBRA HA and NA vaccines with CpG1018 or no adjuvant. Sera were collected pooled following the second vaccination at days 42 and 48 from naïve mice and days 76 or 83 from pre-immune mice and tested for HAI activity against (**A**) three H1N1 viruses, Cal/09, Bris/18, and GD/19 or (**B**) six H3N2 influenza viruses, Swit/13, HK/14, Sing/16, KS/17, HK/19, and SA/19. Represented on the y-axis are the HAI titers on a log 2 scale. Represented on the x-axis are the panels of H1N1 and H3N2 influenza viruses. The top dotted line indicates a 1:80 HAI titer, and the bottom dotted line indicates a 1:40 HAI titer. Each column represents the average +/− standard error of the mean (SEM) of 18 (IM naïve), 14 (IM pre-immune), or 10 (IN pre-immune) individual mice. Each dot represents the HAI titer for each individual mouse. HAI titers were analyzed for statistical relevance using nonparametric one-way analysis of variance (ANOVA) by Prism 9 software (GraphPad Software, Inc., San Diego, CA, USA, version 9.4.0, https://www.graphpad.com). *p* value < 0.05 was defined as statistically significant. *p* < 0.01 **, *p* < 0.0001 ****.

Serum samples collected from naïve mice vaccinated intramuscularly with COBRA vaccine with or without CpG had, on average, HAI titers ≤ 1:40 against all the viruses with ~50% of the mice seroconverting against the CA/09 and BR/18 H1N1 influenza viruses (Figure 5A). All mice had low to no detectable serum HAI activity against any of the H3N2 influenza viruses tested (Figure 5B). In contrast, pre-immune mice vaccinated intramuscularly with COBRA vaccines with or without CpG 1018 adjuvant had, on average, HAI titers > 1:40 against all the H1N1 influenza viruses and 50% of the H3N2 influenza viruses. Interestingly, pre-immune mice vaccinated intranasally with COBRA vaccines without adjuvant had, on average, HAI titer ≥ 1:80 against the H1N1 influenza viruses, with all mice seroconverting to the CA/09 and BR/18 viruses and 75% of mice seroconverting to the GD/19 influenza virus (Figure 5A). Pre-immune mice vaccinated intranasally with the COBRA vaccine plus CpG 1018 had high HAI titers that ranged, on average, from 1:160 to 1:640 against the H1N1 influenza viruses and HAI titers against the H3N2 viruses were, on average, ≥1:40 against all viruses, except HK/19 (Figure 5B).

Naïve mice vaccinated intramuscularly with the COBRA vaccine without adjuvant had NAI activity against both the Bris/18 H1N1 influenza virus and the Swit/13 H3N2 influenza virus (Table 1). There was little to no NAI activity detected in naïve mice vaccinated intramuscularly with the COBRA vaccine with CpG 1018 adjuvant. Pre-immune mice vaccinated intramuscularly or intranasally with these vaccines with or without CpG 1018 adjuvant had similar NAI activity regardless of the route of inoculation or administration of CpG 1018 adjuvant. All NAI activities in the pre-immune mice were within 2–2.5 fold of each other (Table 1).

### 3.2. CpG 1018 Adjuvant Drives Production of IgG and IgA Antibody-Secreting Cells Following Vaccination

**Figure 6:**
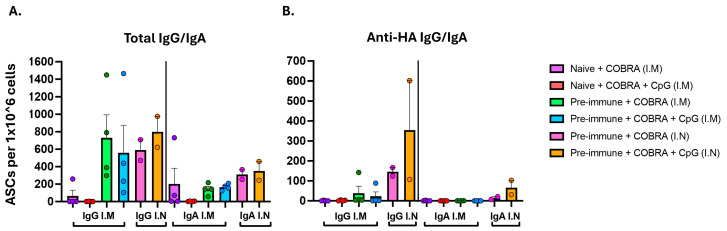
HA-specific IgG and IgA antibody-secreting cells (ASCs) from vaccinated mice. Mice were vaccinated with COBRA HA and NA vaccines with CpG1018 or no adjuvant. Spleens were harvested from naïve mice at day 48 following the booster vaccination and from pre-immune, IM-vaccinated mice at day 6 following the prime vaccinations. In addition, spleens were collected from pre-immune vaccinated IN mice at day 35. Plates were coated with anti-Igκ/λ for measuring (**A**) total IgG or IgA ASCs or coated with (**B**) WT Bris/18 rHA for measuring influenza virus-specific IgG or IgA ASCs. The y-axis represents the number of ASCs per one million input cells collected from each mouse.

Spleens were harvested 20 days (D:48) post-boost (naïve mice), 6 days following one vaccination (IM pre-immune mice), or 7 days following two vaccinations (IN pre-immune mice) to determine the number of ASCs (Figure 6). Spleens of naïve mice vaccinated IM with COBRA HA/NA vaccines with CpG 1018 or with no adjuvant had little to no total IgG or IgA ASCs (Figure 6A) and had no IgG or IgA ASCs that were specific to WT Bris/18 rHA (Figure 6B). Spleens of pre-immune mice IM vaccinated with COBRA HA/NA vaccines with CpG 1018 or with no adjuvant had total non-specific IgG and IgA ASCs but low to no specificity to WT Bris/18 rHA or Bris/18 rHA (Figure 6A,B). In contrast, spleens collected from pre-immune mice vaccinated IN with COBRA HA/NA vaccines with CpG 1018 had enhanced numbers of IgG and IgA ASCs that were specific to the Bris/18 rHA compared to ASCs collected from mice vaccinated with unadjuvanted vaccines (Figure 6B).

**Figure 7:**
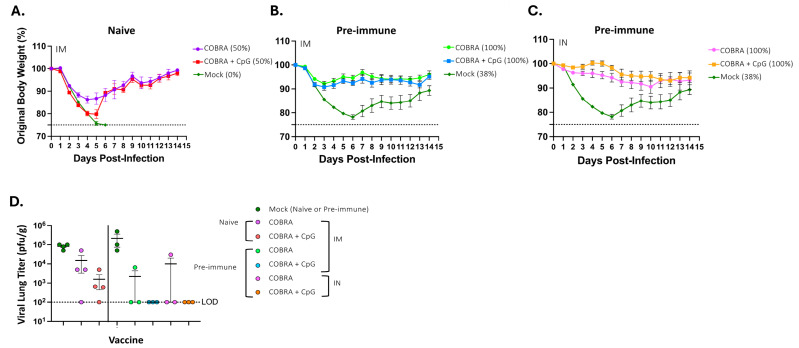
Percent of original mouse weight following influenza virus challenge. All vaccinated and mock vaccinated mice were challenged intranasally with the H1N1 strain, A/Brisbane/02/2018 (8 × 10^6^ PFU/50 μL), and observed for 14 days post-infection. (**A**) Percent of original body weight of naïve mice IM vaccinated, (**B**) percent of original body weight loss of pre-immune mice IM vaccinated, and (**C**) percent of original body weight loss of pre-immune mice IN vaccinated. The dotted line in (**A**–**C**) represents the 75% of original weight endpoint cutoff. Each line (**A**–**C**) represents the average +/− standard error of the mean (SEM). (**D**) Mouse viral lung titers were assessed for three following challenges with A/Brisbane/02/2018 (H1N1) influenza virus in naïve and pre-immune mice. The y-axis represents post-challenge lung viral titers (PFU/g of tissue), and the x-axis represents the vaccine doses. The dotted line represents the limit of detection (LOD).

Mice were challenged at 28 days after the last vaccination with Bris/18 H1N1 influenza virus (8 × 10^6^ PFU/50 μL) (Figure 7). Unvaccinated naïve mice rapidly lost weight and showed signs of infection, including lethargy, and reached 75% of their original weight by day 6 post-infection and were euthanized (Figure 7A). These mice had high viral lung titers (~1 × 10^5^ pfu/g) on day 3 post-infection (Figure 7D). Naïve mice vaccinated intramuscularly with COBRA vaccines with no adjuvant had similar weight loss as unvaccinated mice and reached 80% of their original weight by day 4 post-infection, with 50% of mice not surviving challenge. These mice had an average half-log lower viral lung titer than unvaccinated mice with undetectable viral lung titers in one mouse. The remaining mice in the group began to gain weight and recover from infection. Naïve mice vaccinated intramuscularly with COBRA vaccines with CpG 1018 lost less weight on average than mice vaccinated with COBRA vaccine only. Mice in this group had lost, on average, 12% of their weight by day 4 post-infection, with 50% of mice surviving challenge and the remaining mice recovering to their original weight by day 14 post-infection. These mice had an average viral lung titer of ~3.3 × 10^3^ pfu/g on day 3 post-infection (Figure 7D).

Unvaccinated pre-immune mice also rapidly lost weight by day 6 post-infection, with 38% of mice surviving challenge (Figure 7B,C) with an average viral lung titer of ~5.5 × 10^5^ pfu/g on day 3 post-infection (Figure 7D). Pre-immune mice vaccinated intramuscularly with the COBRA vaccines with or without CpG 1018 lost 5–7% of their weight by day 2 post-challenge, and the mice slowly gained weight over the 14 days of observation, with 100% of mice surviving challenge (Figure 7B). Pre-immune mice vaccinated intranasally with the COBRA vaccines with or without CpG 1018 lost 5–7% of their original weight by day 10 post-challenge and then maintained their weight for the remaining 4 days of observation with all mice surviving challenge (Figure 7C). Viral titers were undetectable in 10 of the 12 collected lungs from these four pre-immune vaccinated mouse groups, and the two mice with detectable viral lung titers were vaccinated with COBRA vaccines without adjuvant. All pre-immune mice vaccinated with COBRA vaccines plus CpG 1018 had no detectable viral lung titers at day 3 post-infection, regardless of whether they were vaccinated intramuscularly or intranasally (Figure 7D).

## 4. Discussion

Our group has published on the use of broadly reactive influenza HA and NA antigens produced using computationally optimized broadly reactive antigen (COBRA) methodology to elicit antibodies that protect against a broad panel of influenza viruses per subtype [7,9,10,11]. To assess the ability of CpG oligodeoxynucleotide sequences to enhance the COBRA HA and NA elicited antibody responses, purified recombinant HA and NA proteins were mixed with CpG 1018 and used to vaccinate mice. This vaccine-adjuvant combination was administered either by intramuscular injection or by intranasal inoculation. The use of unadjuvanted vaccines often does not induce adequate immune responses, particularly when recombinant proteins are administered intranasally [5].

Currently, the oil-in-water emulsion (MF59) adjuvant formulated in the FLUAD vaccine (Seqirus Inc., Holly Springs, NC, USA) is administered intramuscularly and induces protective antibodies in the sera of vaccinated elderly patients. Several novel adjuvant systems are approved for use with vaccines in clinical trials, including CpG 1018 and Alum [15,19]. However, parentally administered vaccines mainly induce systemic immune responses, whereas intranasally administered vaccines have the potential to induce both systemic and mucosal immune responses. This is a characteristic that is effective against pathogens, such as influenza viruses, that naturally infect via mucosal compartments of the respiratory tract [20]. CpG 1018 is a short 22-mer oligonucleotide sequence containing CpG motifs in rodents, non-human primates, and humans that activates TLR9 within-host immune cells, including dendritic cells, natural killer cells, macrophages, and B cells, depending on species [21]. Additionally, this adjuvant induces Th1 and Th2 pro-inflammatory immune responses associated with IFN-γ via the MyD88 pathway [22,23].

CpG ODNs-induced Th1-biased immune responses are associated with cytotoxic T cell activity that kills and clears infected cells following infections when neutralizing antibodies are not present [24]. However, Th1 CD4+ T cell responses also induce the activation of B cells that secret opsonizing antibodies that are able to mark infected cells for killing during an active viral infection [25]. CpG 1018 promotes IgG2a/2b isotype responses but also often increases IgG1 response levels as well. Mice immunized with COBRA HA/NA protein plus CpG 1018 have enhanced IgG2a/IgG2b Th1 biased responses compared to vaccine-only immunized mice, resulting in a more balanced IgG1:IgG2a T helper response observed in this study. Additionally, the elicitation of low HAI and NAI activities by vaccination in naïve mice resulted in limited protection against influenza virus challenge. The elicited serum antibodies did not correlate with IgG or IgA antibody-secreting cells in the spleens of these mice. Similarly, naïve mice vaccinated IM with COBRA HA/NA vaccines with no adjuvant induced little to no serum antibodies with HAI activity against H1N1 and H3N2 influenza viruses, but there was detectable NAI activity. However, this NAI activity was not sufficient to protect mice from influenza virus infection. These low serum antibody titers were in line with the number of splenic IgG antigen presenting cells. Naïve mice that were vaccinated IM with COBRA HA/NA vaccines with or without CpG had little to no neutralizing antibodies and, therefore, the influenza challenge viruses infected host cells. In turn, this may have stimulated CpG-dependent CTL responses [9] that were directed towards the infected cells. In addition, the production of opsonizing antibodies may not have been sufficient to eliminate, only reduce, viral infection, which resulted in 50% of the mice surviving challenge. Moreover, COBRA NA-induced antibodies, although low in these mice, may have also contributed to some level of reduction of the viral lung titers following challenge by targeting infected cells for killing or blocking the NA enzyme activity from cleaving progeny virions.

Mice that were pre-immunized with the historical H1N1 and H3N2 influenza viruses and then vaccinated with COBRA HA/NA vaccines with or without adjuvant all had enhanced HAI activity and NAI titers, especially the pre-immune mice that were vaccinated intranasally. These mice had anti-HA and anti-NA serum IgG following vaccination. The level of vaccine-induced IgG antibodies is related to the pre-existing immunity via follicular CD4^+^ T cells [26]. Enhanced IgG titers were also observed in the BALFs of mice vaccinated intranasally. Notably, in pre-immune mice that were vaccinated IM, the use of CpG 1018 induces a balanced Th1/Th2 immune response compared to unadjuvanted vaccines, which was similar to that in naïve IM vaccinated mice. Furthermore, unlike naïve mice vaccinated IM or pre-immune mice vaccinated IN, the pre-immune mice vaccinated IM with COBRA HA/NA vaccines with CpG 1018 induced enhanced serum IgA antibodies, suggesting that CpG 1018 induces additional immune mediators by various routes of administration, such as mucosal IgA secreting B cells that maintain the integrity of the mucosal barriers [27]. Additional beneficial outcomes by formulating with CpG 1018 were observed in pre-immune mice that were vaccinated IM with COBRA HA/NA vaccines with CpG, having a more balanced Th0 profile, whereas pre-immune mice that were vaccinated IM with COBRA HA/NA vaccines without adjuvant had a Th2 biased immune responses. Pre-immune mice vaccinated with either IM or IN also had both IgG and IgA antigen presenting cells detected in the spleen.

All vaccinated pre-immune mice survived influenza virus infection. However, ~38% of the unvaccinated but pre-immune mice also survived influenza virus challenge. This may be due to cross-reactive immune responses, both antibodies and T cells, elicited by the historical influenza viruses used to establish memory responses [28]. Pre-existing immune responses elicited by historical influenza virus strains imprint on the memory cell population and shape the outcomes of subsequent vaccinations, possibly through the overall memory response of previous exposure to influenza viruses, which is translated to protection against influenza virus infection. Antibody responses are generally short-lived in naïve mice since they fail to maintain optimal B cell pools in the bone marrow during early life [29]. Specifically, post-germinal plasmablast B cells are home to bone marrow, but since naïve mice have little to no differentiation survival signals, they remain immunologically incompetent [29]. In contrast, multiple exposures to various antigens throughout life leads to an overexpression of different classes of antibodies derived from B cell clones and increase the pool of organ-specific and organ-non-specific autoantibodies [29]. Administering CpG ODNs drives the induction of dendritic and T cell activation patterns, as well as the activation of pre-existing memory B cells [29,30]. It is unclear whether Th1- or Th2-biased immune responses are most effective against influenza virus infection [31]; however, the findings in this study indicated that in immunologically naïve mice, the combination of low serum HAI and NAI activity and a balanced IgG isotype profile were not sufficient to protect mice against influenza virus-induced disease and death. However, vaccinating pre-immune mice via IM or IN route with vaccine plus CpG 1018 adjuvant did induce both IgG, IgA, and a broader range of isotypes (IgG1, IgG2a, IgG2a) [24].

Overall, these findings suggest that adjuvating COBRA HA/NA vaccines with CpG 1018 enhanced broadly reactive antibodies. The effectiveness of the induced immune responses by these vaccines in mice with pre-existing immunity to influenza viruses may resemble the situation in people who have memory cells from a lifetime of influenza vaccinations and infections. In contrast, naïve mice have no immune responses to influenza viruses, which resembles the infants and young children who have yet to be exposed to influenza viruses. In both scenarios, broadly reactive HAI and NAI activity against several drifted H1N1 and H3N2 influenza viruses were elicited by this vaccine formulation compared to unadjuvanted COBRA HA/NA vaccines. Furthermore, vaccinating pre-immune mice IN with COBRA HA/NA vaccines adjuvanted with CpG 1018 induced effective influenza virus-specific mucosal and systemic responses and protected mice from influenza virus infection. Specifically, since the nasal mucosa has a wider vesiculated surface area, COBRA HA/NA vaccine mixed with CpG 1018 may have been absorbed through the local mucosal lymphoid follicles referred to as nasal-associated lymphoid (NALT). This area of vaccination is beneficial since this is the site where CpG 1018 and COBRA HA/NA antigens first reach the respiratory tract [32]. This may also suggest that IN administration of CpG 1018 in these pre-immune models could also recall resident mucosal CTLs that were initially induced by prior influenza virus exposure. Therefore, mice with pre-existing immune responses to influenza viruses that were vaccinated intranasally with vaccine and CpG 1018 elicited local CTLs [33] or locally distinct CpG-NALT-associated responses [34]. In turn, these findings offer an option for the development of COBRA HA/NA vaccines that can potentially be tailored with CpG 1018 adjuvant for inducing distinct adjuvant profiles that may be relevant for pre-immune populations since people have diverse memory immune history to previous influenza viruses.

## 5. Conclusions

Overall, COBRA HA/NA vaccines elicit broadly-reactive immune responses against H1N1 and H3N2 influenza viruses that are enhanced by CpG1018 adjuvant following intranasal delivery. This vaccine and adjuvant combination provides an efficient and effective method to stimulate protective immunity.

## Figures and Tables

**Table 1:** Neuraminidase inhibition (NAI) titer.

Vaccine	Immune Status	Route	Bris/18 NA	Swit/13 NA
COBRA	Naïve	Intramuscular	7.80	9.00
COBRA + CpG	Naïve	Intramuscular	3.50	2.20
COBRA	Pre-immune	Intramuscular	10.00	11.25
COBRA + CpG	Pre-immune	Intramuscular	8.75	11.00
COBRA	Pre-immune	Intranasal	8.20	10.10
COBRA + CpG	Pre-immune	Intranasal	9.00	9.00

Reciprocal value of the neuraminidase inhibition (NAI) titer of pooled serum samples collected 14 days post-boost per vaccine group against the H1N1 influenza virus A/Brisbane/02/2018 and the H3N2 influenza virus A/Switzerland/9715293/2013.

## Data Availability

The data are contained within the article.
